# Low absolute peripheral blood CD4+ T-cell count predicts poor prognosis in R-CHOP-treated patients with diffuse large B-cell lymphoma

**DOI:** 10.1038/bcj.2017.37

**Published:** 2017-04-21

**Authors:** Y Kusano, M Yokoyama, Y Terui, N Nishimura, Y Mishima, K Ueda, N Tsuyama, Y Hirofumi, A Takahashi, N Inoue, K Takeuchi, K Hatake

**Affiliations:** 1 Department of Hematology Oncology, Hematology Oncology, Cancer Institute Hospital, Japanese Foundation for Cancer Research, Tokyo, Japan; 2 Division of Pathology, The Cancer Institute, Japanese Foundation for Cancer Research, Tokyo, Japan; 3 Pathology Project for Molecular Targets, The Cancer Institute, Japanese Foundation for Cancer Research, Tokyo, Japan

## Abstract

The absolute peripheral blood lymphocyte count at diagnosis is known to be a strong prognostic factor in patients with diffuse large B-cell lymphoma (DLBCL) treated with rituximab, cyclophosphamide, doxorubicin, vincristine and prednisone (R-CHOP), but it remains unclear as to which peripheral blood lymphocyte population is reflective of DLBCL prognosis. In this cohort, 355 patients with DLBCL treated with R-CHOP from 2006 to 2013 were analyzed. The low absolute CD4+ T-cell count (ACD4C) at diagnosis negatively correlated with the overall response rate and the complete response rate significantly (*P*<0.00001). An ACD4C<343 × 10^6^/l had a significant negative impact on the 5-year progression-free survival and the overall survival as compared with an ACD4C⩾343 × 10^6^/l (73.7% (95% confidence interval (CI)=66.7–79.5) versus 50.3% (95% CI=39.0–60.6), *P*<0.00001 and 83.3% (95% CI=77.1–88.0) versus 59.0% (95% CI=47.9–68.5), *P*<0.00000001, respectively). Multivariate analysis revealed that the ACD4C was an independent prognostic marker (hazard ratio=2.2 (95% CI=1.3–3.7), *P*<0.01). In conclusion, a low ACD4C at diagnosis served as an independent poor prognostic marker in patients with DLBCL.

## Introduction

Rituximab, cyclophosphamide, doxorubicin, vincristine and prednisone (R-CHOP) therapy is indicated for diffuse large B-cell lymphoma (DLBCL) patients regardless of the revised International Prognostic Index (IPI).^1, 2^ Although the 3-year overall survival (OS) in the poor-risk group in revised IPI was 60%,^2^ it has been known that patients with DLBCL had more severe prognosis, suggesting a need for other useful prognostic markers at diagnosis. An absolute lymphocyte count (ALC) is known to be a strong prognostic factor in DLBCL that is the most common non-Hodgkin lymphoma type. A low ALC, and high absolute monocyte count (AMC) in peripheral blood before and after R-CHOP therapy can predict short-term survival in DLBCL^3, 4, 5, 6, 7, 8, 9, 10, 11, 12, 13^ and a low ALC is also known to negatively affect survival, not in germinal center (GC)-type DLBCL but in non-GC-type according to Hans algorithm.^7, 14^ Currently, tumor-specific CD4+ T cells are known to have a role in the immune response against cancer, because CD4+ T cells facilitate the activation of cytotoxic CD8+ T cells and the antitumor effects of CD8+ T cells are limited in the absence of CD4+ T cells.^15, 16^ In addition, CD4+ T cells can also target cancer cells in the absence of CD8+ T cells.^17, 18^ Abnormalities in the CD4+ T-cell compartment, including the Th1 and Th2 subsets and related cytokines, have been shown to affect the occurrence of non-Hodgkin lymphoma, demonstrating that CD4+ T cells exert the strongest immunological response against tumors.^19, 20^ On the other hand, it is not known whether peripheral blood T-cell subsets interfere with the prognosis of DLBCL. Recently, the ACD4C was identified as a prognostic marker in indolent lymphomas.^21, 22^ Even in DLBCL, ACD4C was suggested as a prognostic marker in patients with R-CHOP.^23^ To further investigate the relationships of the host immunity at diagnosis with survival of DLBCL patients after R-CHOP and with other prognostic factors of DLBCL are crucial factors for improving the standard therapy in DLBCL. Thus, we primarily investigated the relationships of the pretreatment absolute lymphocyte subset counts with survival and other prognostic factors in *de novo* DLBCL patients.

## Patients and Methods

### Patients

All patients included in this study were older than 18 years and they were histologically diagnosed with untreated *de novo* DLBCL by expert hematopathologists according to the World Health Organization classification,^24^ at a single cancer institute between 2006 and 2013. The cell of origin of DLBCL was assessed using the Hans algorithm.^25^ Patients were excluded if they had DLBCL secondary to non-Hodgkin lymphoma, clinically relevant heart disease or antibodies against human immunodeficiency virus. All patients provided informed consent to receive treatment and written informed consent allowing the use of their medical records. The review boards at our institute approved a retrospective review of medical records, which was conducted in accordance with the Declaration of Helsinki. Patients were staged according to the Ann Arbor classification.^26^ All patients underwent a physical examination, lymph node biopsy and bone marrow aspiration and biopsy. The disease stage was assessed using enhanced computerized tomography scans from the neck to the pelvis and whole-body positron emission tomography scans. The following clinical and laboratory data were available at the time of diagnosis: age, gender, ALC, absolute B-cell count, ACD4C, absolute CD8+ T-cell count (ACD8C), AMC, serum lactate dehydrogenase (LDH), serum albumin, performance status (PS), B symptoms, clinical stage, β2-microglobulin level, soluble IL-2 receptor and bone marrow involvement. Lymphocyte subset counts were calculated from the percentages obtained by flow cytometry.^27^ All data were collected and recorded in a computerized database.

### Treatment

All patients were scheduled to receive six to eight full cycles of R-CHOP-21 therapy.^28^ Responses were determined according to the International Workshop criteria.^29^

### Statistical analysis

Survival endpoints were evaluated using the Kaplan–Meier method and Cox proportional hazard model. Optimal cutoff values of other lymphocyte populations at diagnosis were determined on the basis of the utility of these parameters as markers of survival using receiver operating characteristic (ROC) curves. Fisher's exact test was used to determine the relationships between the absolute number of lymphocyte subsets and the prognostic factors of DLBCL evaluated as nominal variables. Pearson's correlation coefficients were used to evaluate the association between the absolute number of lymphocyte subsets and the continuous variables known as prognostic factors of DLBCL. Differences between the results of comparative tests were considered significant if the two-sided *P*-value was<0.05. EZR v3 was used for the statistical analysis.^30^

## Results

### Patients

A total of 414 patients were diagnosed with DLBCL at our institute from 2006 to 2013. Three hundred and fifty-five patients with *de novo* DLBCL were included in the analysis (Table 1). The median patient age was 65 years (range: 20–89 years) and 243 patients (68%) were older than 60 years. The male/female ratio was 1.2:1. In addition, 19 patients (5%) had an Eastern Cooperative Oncology Group PS⩾2 and 152 patients (43%) had elevated LDH levels. A total of 145 patients (41%) had Ann Arbor stage⩾3 disease and⩾2 extranodal sites were involved in 93 patients (26%). The median values of ALC, ACD4C, ACD8C and AMC were 1310 × 10^6^/l (range 100–7080 × 10^6^/l), 459 × 10^6^/l (range 11–1944 × 10^6^/l), 338 × 10^6^/l (range 3–1518 × 10^6^/l) and 372 × 10^6^/l (range 0–5065 × 10^6^/l), respectively. Regarding lymphocyte populations, 198 patients (56%) had a low ALC (<1380 × 10^6^/l), 113 (32%) had a low ACD4C (<343 × 10^6^/l), 68 (19%) had a low ACD8C (<191 × 10^6^/l) and 107 (30%) had a high AMC (<529 × 10^6^/l). GC DLBCL was diagnosed in 167 patients (53%), and non-GC DLBCL was observed in 148 patients (47%). Additionally, 26 patients (7%) received addition of radiotherapy after R-CHOP.

The overall response rate was 95% in the low IPI group (score=0–2) and 88% in the high IPI group (score=3–5). After a median follow-up of 57 months, the 5-year progression-free survival (PFS) and OS rates in the low IPI group were 76.0% and 83.7%, respectively, whereas those in the high IPI group were 41.8% and 55.4%, respectively. The 5-year PFS and OS in the GC group were 74.4% and 81.8%, respectively, whereas the corresponding rates in the non-GC group were 57.4% and 69.9%, respectively.

### ALC at diagnosis

**Figure 1:**
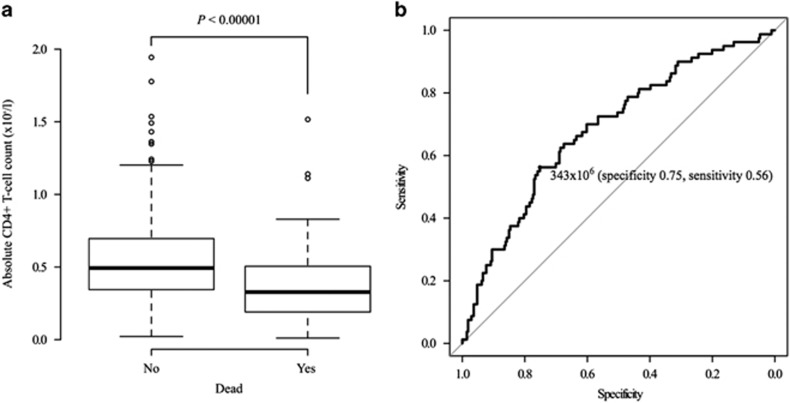
Box plot analysis and area under the curve analysis for the ACD4C in the present study. (**a**) Box plot analysis of the ACD4C before R-CHOP therapy in patients who had died or remained alive till the last follow-up. The median pretreatment ACD4C was significantly higher among survivors than among deceased patients (544 × 10^6^/l versus 375 × 10^6^/l, *P*<0.00001). (**b**) ROC curve evaluating ACD4C at diagnosis as a marker for death after R-CHOP treatment. Area under the curve (AUC) analysis revealed a cut-off value of 343 × 10^6^/l (AUC=0.68, specificity 0.75, sensitivity 0.56).

First, box plot analysis illustrated that patients survived till the last follow-up after R-CHOP treatment had a higher ALC, ACD4C, ACD8C and AMC than those who died during the study period (blot plot of ACD4C is shown in Figure 1a, the median ACD4C was 544 × 10^6^/l (range: 237 × 10^6^–845 × 10^6^/l, *n*=275) among surviving patients versus 375 × 10^6^/l (range: 115 × 10^6^–619 × 10^6^/l, *n*=80) among patients who died (*P*<0.00001)). The absolute B-cell count before R-CHOP did not differ between surviving and deceased patients according to a box plot analysis, and no further evaluation was performed. Second, to determine the cutoff value for evaluating individual lymphocyte subsets at diagnosis, we performed area under the ROC curve analysis and then the optimal ALC, ACD8C and AMC cutoff values were determined as 1380 × 10^6^, 191 × 10^6^ and 549 × 10^6^/l as negative survival markers, respectively (data not shown). In terms of ACD4C, ROC curve showed 343 × 10^6^/l as a cutoff value (area under the ROC curve=0.69, Figure 1b). Thus, these values were chosen as the optimal cutoff value and were used in all subsequent analyses in the present study. ALC had strong correlations not only with ACD4C (Pearson's *R*-value=0.76, *P*=0, Figure 3a) but also with ACD8C (Pearson's *R*-value=0.68, *P*=0) using Pearson's correlation coefficients.

### Therapeutic outcomes according to the ALC, ACD8C, AMC and ACD4C

**Figure 2:**
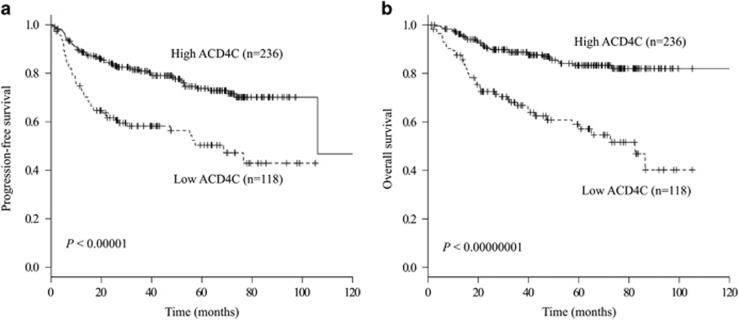
Clinical outcomes of patients according to the ACD4C at diagnosis. (**a**) PFS. (**b**) OS. Both survival rates were higher in the high ACD4C group than in the low ACD4C group (*P*<0.0001 for PFS and *P*<0.00000001 for OS).

The overall response rate was higher in patients with high ALCs than those with low ALCs. The overall response rate was 96% in the high ALC group versus 84% in the low ALC group (*P*<0.001), 97% in the high ACD4C group versus 74% in the low ACD4C group (*P*<0.00000001) and 94% in the high ACD8C versus 72% in the low ACD8C (*P*<0.00001), whereas the overall response rate was not dependent on AMC (91% in the low AMC group versus 86% in the high AMC group, *P*=0.18). Similar trends were observed in the achievement of the complete response rate for the ALC (90% in the high ALC group versus 82% in the low ALC group, *P*=0.04) and ACD4C (92% in the high ACD4C group versus 73% in the low ACD4C group, *P<*0.0001), and ACD8C (89% in the high ACD8C group versus 72% in the low ACD8C group, *P<*0.001), whereas the complete response rate was not dependent on AMC (87% in the low AMC group versus 82% in the high ACD8C group, *P*=0.23). The estimated PFS and OS rates were significantly different between high and low lymphocyte subsets. Kaplan–Meier analyses yielded the 5-year PFS rates of 76.5% (95% confidence interval (CI)=68.0–82.9) and 57.8% (95% CI=49.3–65.5) in the high and low ALC groups (*P*<0.0001), 73.7% (95% CI=66.7–79.5) and 50.3% (95% CI=39.0–60.6) in the high and low ACD4C groups (Figure 2a, *P*<0.00001), 71.0% (95% CI=64.5–76.6) and 46.1% (95% CI=31.3–59.7) in the high and low ACD8C groups (*P*<0.00001), and 56.9% (95% CI=63.0–76.0) and 70.1% (95% CI=45.2–67.0) in the high and low AMC groups (*P*=0.01), respectively. Furthermore, the 5-year OS rates were 85.8% (95% CI=78.4–90.8) and 67.1% (95% CI=58.9–74.1) in the high and low ALC groups (*P*<0.0001), 83.3% (95% CI=77.1–88.0) and 59.0% (95% CI=47.9–68.5) in the high and low ACD4C groups (Figure 2b, *P*<0.00000001), 80.2% (95% CI=74.4–84.8) and 55.6% (95% CI=40.1–68.7) in the high and low ACD8C groups (*P*<0.00001), and 68.8% (95% CI=72.0–83.5) and 78.4% (95% CI=57.4–77.7) in the high and low AMC groups (*P*=0.07), respectively.

### Univariate and multivariate Cox hazard regression analyses

Low ACD4C, low ACD8C, high AMC and other subgroups were subjected to Cox hazard regression analyses, to further investigate the prognostic values of these parameters. Low ALC was excluded from the Cox hazard regression, because ALC had positive correlations with ACD4C and ACD8C as mentioned above. Univariate analyses of OS prognostic factors after R-CHOP treatment indicated that low ACD4C and low ACD8C at diagnosis correlated significantly with clinical outcomes (Table 2). Other significant factors associated with OS after R-CHOP therapy in the univariate setting included age <60 years, Eastern Cooperative Oncology Group PS⩾2, elevated LDH levels, ⩾2 sites of extranodal disease involvement, stage III/IV disease and high IPI (score 3–5). High AMC and non-GC was not associated with poor OS in this cohort.

Multivariate analysis 1 included elevated LDH, ⩾2 sites of extranodal disease involvement, age>60 years, stage III/IV, PS>2 (all IPI factors), non-GC type, low ACD4C, low ACD8C and high AMC. ACD4C<350 × 10^6^/l at diagnosis remained as a significant prognostic factor (hazard ratio (HR)=2.2, 95% CI=1.3–3.7, *P*<0.01, Table 2). Multivariate analysis 2 included high IPI, non-GC type, low ACD4C, low ACD8C and high AMC. The ACD4C<350 × 10^6^/l at diagnosis was associated with an adjusted HR for OS after R-CHOP of 2.3 (95% CI=1.4–3.9, *P*<0.01, Table 2). Low ACD8C and high AMC were not associated with poor OS in multivariate analysis.

### Influence of ACD4C in baseline patient characteristics

**Figure 3:**
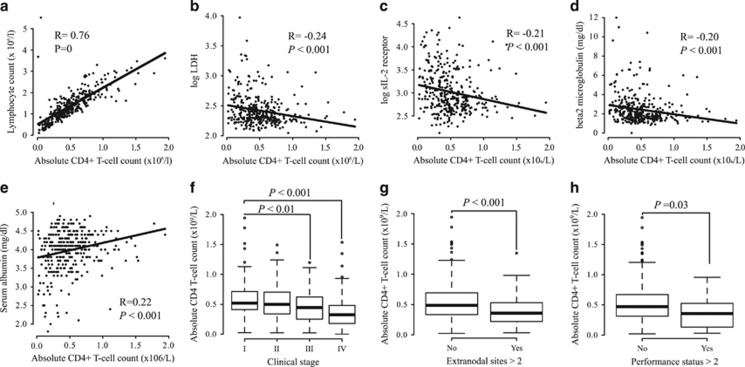
Correlations between the ACD4C and poor prognostic factors. (**a**) A strong correlation was identified between ALC and ACD4C (Pearson's *R*-value 0.76, *P*=0). (**b–d**) Negative correlations were identified between the ACD4C and the log-normal distribution of the LDH level (log_10_ LDH), log-normal distribution of the soluble IL-2 receptor level (log_10_ sIL-2 receptor) and β2-microglobulin level. Pearson's *R*-values were −0.24 (*P*<0.001), −2.0 (*P*<0.001), −2.1 (*P*<0.001) and −2.2 (*P*<0.001), respectively, for these variables. (**e**) The ACD4C decreased in proportion to the albumin level. (**f–h**) Box plot analysis revealed significant negative correlations of the ACD4C with the Ann Arbor stage (*P*<0.001), extranodal disease involvement (*P*<0.001) and PS (*P*=0.03).

We identified correlations between ACD4C and known prognostic markers such as LDH, sIL-2R,^31^ β2-microglobulin and albumin using Pearson's correlation coefficients (LDH (Pearson's *R*-value was −0.24, Figure 3b), sIL-2R (Pearson's *R*-value was −0.21, Figure 3c) and β2 microglobulin (Pearson's *R*-value was −0.20, Figure 3d)). In addition, we also identified relationships of the ACD4C with clinical stage, PS and extranodal disease using box plot analysis (clinical stage (The mean ACD4C in stage I was 602 or significantly higher than that in stage III (471, *P*<0.01) or stage IV (377, *P*<0.00001). The mean ACD4C values differed significantly between stage III and stage IV (*P*=0.04), Figure 3e), extranodal disease (The mean ACD4C values were 0.54 in <2 involved extranodal sites versus 0.4 in ⩾2 involved extranodal sites (*P*<0.001), Figure 3f) and PS (The mean ACD4C values were 0.51 in Eastern Cooperative Oncology Group PS<2 versus 0.36 in Eastern Cooperative Oncology Group PS⩾2 (*P*=0.03), Figure 3g)). Variables that increased with an increase in the disease progression had inverse correlations with the ACD4C, whereas albumin, the levels of which declined with disease progression, had a positive correlation with the ACD4C (Pearson's *R*-value was 0.22, Figure 3d).

### Survival according to the ACD4C combined with poor prognostic factors

**Figure 4:**
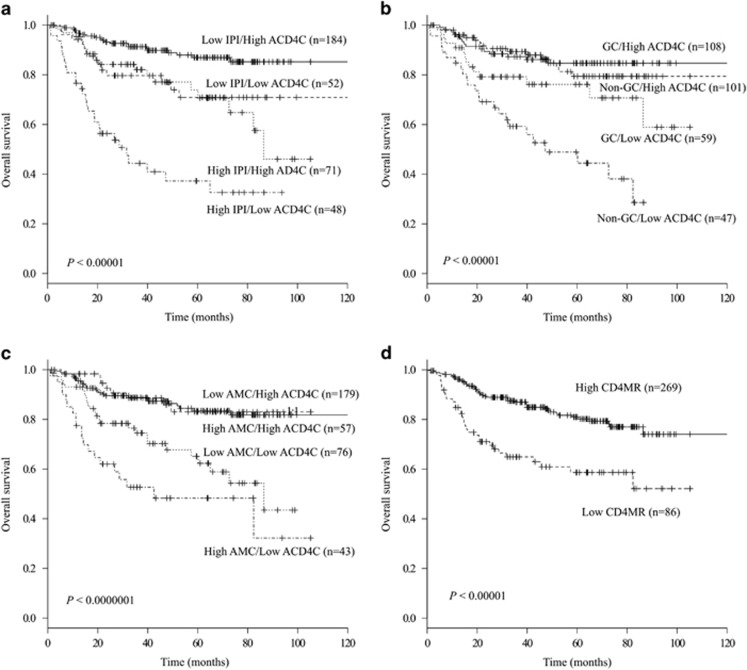
The therapeutic outcomes according to prognostic factors and the ACD4C. (**a**) OS was dependent on ACD4C in either the low or high IPI group but ACD4C strongly affected survival in the high IPI group (*P*<0.001 and *P*<0.0001 for the low and high IPI groups, respectively). (**b**) According to the Hans criteria, the OS differed with respect to ACD4C significantly in either GC B-cell (GC)-type (*P*=0.04) or non-GC type (*P*<0.000001). ACD4C strongly affected survival in the non-GC group. (**c**) OS was significantly lower in the low ACD4C group, despite the AMC was low (*P*<0.001) or high (*P*<0.001), compared with the high ACD4C group. High AMC tended to affect survival in the low ACD4C (*P*=0.07), but high AMC did not affect OS in the high ACD4C group in this cohort (*P*=0.94). (**d**) Low ACD4C to AMC ratio (CD4MR<0.64) was associated with significant poor OS (*P*<0.00001).

We investigated the influence of ACD4C at diagnosis combined with IPI, cell-of-origin or AMC for DLBCL patients. Kaplan–Meier curves revealed that low ACD4C showed its influence in survival only in DLBCL patients with high IPI or non-GC. A low ACD4C affected survival outcomes in both the low and high IPI groups. The 5-year OS rates were 86.9% (95% CI=80.2–91.5), 73.9% (95% CI=58.9–84.1), 70.9% (95% CI=54.6–82.1) and 37.2% (95% CI=21.9–52.6) in the high ACD4C/low IPI, lowACD4C/low IPI, high ACD4C/high IPI and low ACD4C/high IPI groups (Figure 4a, *P*<0.00001), respectively. The OS was dependent on ACD4C in either the low or high IPI group (*P*<0.001 and *P*<0.0001 for the low and high IPI groups, respectively). The 5-year OS rates were 84.7% (95% CI=75.8–90.5), 79.4% (95% CI=68.1–87.1), 76.2% (95% CI=61.4–85.9) and 48.9% (95% CI=32.2–63.7) in the high ACD4C/GC, high ACD4C/non-GC, low ACD4C/GC and low ACD4C/non-GC groups (Figure 4b, *P*<0.000001), respectively. The OS differed with respect to the ACD4C significantly not only among patients with GC DLBCL but also among patients with non-GC DLBCL. The OS was significantly lower in the low ACD4C group (*P*=0.04 for GC type and *P*<0.000001 for non-GC type). The 5-year OS rates were 83.4% (95% CI=76.2–88.6), 83.0% (95% CI=68.3–91.3), 65.1% (95% CI=50.9–76.2) and 48.3% (95% CI=30.8–63.8) in the high ACD4C/low AMC, high ACD4C/high AMC, low ACD4C/low AMC and low ACD4C/high AMC groups (Figure 4c, *P*<0.0000001), respectively. OS was significantly lower in the low ACD4C group, despite AMC was low (*P*<0.001) or high (*P*<0.001), compared with the high ACD4C group. High AMC tended to affect survival in the low ACD4C (*P*=0.07), but high AMC did not affect OS in the high ACD4C group in this cohort (*P*=0.94). As mentioned above, the OS was synergistically lower in the low ACD4C group among patients with high IPI, non-GC DLBCL or high AMC, respectively.

We further investigated the influence of the ACD4C to AMC ratio (CD4MR) for DLBCL patients, because lymphocyte to monocyte ratio is known as a prognostic marker in DLBCL.^9, 10, 11, 12, 13, 32, 33, 34, 35^ ROC curve showed the cutoff value of CD4MR of 0.64. The 5-year OS rates were 81.0% (95% CI=74.9–85.7) and 58.7% (95% CI=46.0–69.3) in the high CD4MR and low CD4MR groups (Figure 4d, *P*<0.00001), respectively.

## Discussion

In the present study, we aimed to identify which lymphocyte subsets are most associated with therapeutic outcomes in patients with DLBCL who were treated with R-CHOP. Multivariate analysis illustrated that ACD4C<343 × 10^6^/l had a significant negative impact on the 5-year PFS and OS as compared with ACD4C⩾343 × 10^6^/l. Low ACD8C and high AMC were not independent prognostic factors in this cohort. As ACD4C and ACD8C were strongly correlated with ALC, we excluded ALC from multivariate analysis. A low ACD4C combined with high IPI, non-GC type or high AMC aggravated the survival synergistically, suggesting the importance of synergistic worsening of prognosis due to the influence of these combined factors. Furthermore, low CD4MR affected negative prognosis significantly in DLBCL patients. A cutoff value for the ACD4C was chosen on the basis ROC curve. The initiation of antiretroviral therapy at the level of ACD4C<350 × 10^6^/l is beneficial for human immunodeficiency virus-infected patients, which results in a significant decline in the risk of AIDS-related morbidity and mortality.^36, 37, 38^ These data suggest that ACD4C 340–350 × 10^6^/l is crucial value for human immunity against infection or tumor. Recently, other group measured peripheral ACD4C in 43 DLBCL patients.^24^ In the study, 34 patients (72%) were given R-CHOP and mean value of ACD4C (460 × 10^6^/l) was used as a cutoff value. They documented that low ACD4C was associated with poor PFS using the whole population, whereas ACD4C had an insignificant association with survival in the population treated with rituximab in multivariate analysis, owing to the small numbers. The presumption of Judd's study was that ACD4C predicted survival was validated in the present study. In addition, ALC, ACD8C and natural killer cell counts were insignificantly associated with PFS and OS in the study,^23^ which supports the results of our study.

We further identified the previously unclear relationships of advanced clinical stage, high LDH, low albumin, high β2-microglobulin, ⩾2 sites of extranodal disease and high sIL-2R with a low ACD4C. He *et al.* and Zhang *et al.* described that a low ACD4C was not associated with poor-prognostic markers in patients with follicular lymphoma^21^ and mantle cell lymphoma.^22^ By contrast, a low ACD4C at diagnosis exhibit significant inverse correlations with the prognostic factors in DLBCL, suggesting that an ACD4C correlates with poor prognostic factors only in aggressive B-cell lymphoma.

Retrospective nature is a potential weakness of our study. Thus, further prospective investigations are required in the future, to determine whether the pretreatment ACD4C affects the survival in high-risk DLBCL patients treated with R-CHOP and whether low ACD4C combined with high IPI, non-GC type and high AMC aggravated the survival synergistically.

In conclusion, we demonstrated that pretreatment peripheral ACD4C put a negative impact on survival in DLBCL patients, and that various poor prognostic factors of non-Hodgkin lymphoma were associated with a significant reduction in ACD4C in DLBCL. A low ACD4C at diagnosis had a significant negative impact on the survival of patients with *de novo* DLBCL, who were treated with R-CHOP. A low ACD4C combined with high IPI, non-GC type or high AMC aggravated the survival synergistically in DLBCL.

## Figures and Tables

**Table 1:** Baseline patient characteristics

*Characteristics*	*Values*
Number	355
Median age, years (range)	65 (20–89)
Age >60 years, *n* (%)	243 (68)
Men, *n* (%)	191 (54)
ECOG PS⩾2, *n* (%)	19 (5)
Elevated LDH, *n* (%)	152 (43)
Low ALC (<1380/l), *n* (%)	198 (56)
Low ACD4C (<340/l), *n* (%)	113 (32)
Low ACD8C (<191/l), *n* (%)	68 (19)
GC DLBCL according to Hans criteria, *n* (%)	167 (53)
Ann Arbor stage ⩾3, *n* (%)	145 (41)
⩾2 Involved extranodal sites, *n* (%)	93 (26)
	
*Type of chemotherapy*	
R-CHOP, *n* (%)	355 (100)
+ Radiotherapy, *n* (%)	26 (7)
	
*ORR according to IPI*	
ORR, low IPI (0–2) (*n*=255)	241 (95)
ORR, high IPI (3–5) (*n*=100)	88 (88)
	
*5-Year PFS according to IPI*	
5-Year PFS, low IPI (0–2) (*n*=255)	76.00%
5-Year PFS, high IPI (3–5) (*n*=100)	41.80%
	
*5-Year OS according to IPI*	
5-Year OS, low IPI (0–2) (*n*=255)	83.70%
5-Year OS, high IPI (3–5) (*n*=100)	55.40%

Abbreviations: ABC, absolute B-cell count; ACD4C, absolute CD4+ T-cell count; ACD8C, absolute CD8+ T-cell count; DLBCL, diffuse large B-cell lymphoma; ECOG PS, Eastern Cooperative Oncology Group performance status; GC, germinal center; IPI, International Prognostic Index; ORR, overall response rate; OS, overall survival; PFS, progression-free survival; R-CHOP, rituximab, cyclophosphamide, doxorubicin, vincristine and prednisone.

**Table 2:** Univariate and multivariate analyses of OS predictors

*Variables*	*Univariate*		*Multivariate 1*		*Multivariate 2*	
	*HR (95% CI)*	P-*value*	*HR (95% CI)*	P-*value*	*HR (95% CI)*	P-*value*
Age >60 years	2.0 (1.1–3.5)	0.01	2.2 (1.2–4.2)	0.01		
Male	1.4 (0.9–2.2)	0.16				
ECOG PS ⩾2	4.7 (2.5-8.8)	<0.001	3.0 (1.4–6.1)	<0.01		
Stage III/IV	4.2 (2.6–6.8)	<0.001	3.3 (1.7–6.3)	<0.001		
⩾2 Extranodal sites	2.2 (1.4–3.5)	<0.001	0.9 (0.5–1.6)	0.80		
Elevated LDH	2.3 (1.5–3.6)	<0.001	0.8 (0.5–1.5)	0.52		
Low ACD4C	3.5 (2.2–5.5)	<0.001	2.2 (1.3–3.7)	<0.01	2.3 (1.4–3.9)	<0.01
Low ACD8C	3.0 (1.9–4.8)	<0.001	1.6 (0.9–2.9)	0.10	1.7 (0.9–2.9)	0.08
High AMC	1.5 (1.0–2.4)	0.07	1.3 (0.7–2.2)	0.32	1.2 (0.7–2.0)	0.47
Non-GC type	1.6 (1.0–2.6)	0.05	1.4 (0.8–2.4)	0.18	1.5 (0.9–2.4)	0.13
High IPI	1.8 (1.4–2.1)	<0.01			2.6 (1.6–4.2)	<0.001

Abbreviations: ACD4C, absolute CD4+ T-cell count; ACD8C, absolute CD8+ T-cell count; AMC, absolute monocyte count; DLBCL, diffuse large B-cell lymphoma; ECOG, Eastern Cooperative Oncology Group; GC, germinal center; HR, hazard ratio; IPI, International Prognostic Index; LDH, lactate dehydrogenase; OS, overall survival.
